# Assessing the Efficacy of the FOUR (Full Outline of Unresponsiveness) Score in Diabetic Ketoacidosis-Induced Altered Mental Status

**DOI:** 10.7759/cureus.95745

**Published:** 2025-10-30

**Authors:** Sanjay Kumar Sudhakar, Sahasyaa Adalarasan, Sandhiya T, Yogesh S, Hariharan C

**Affiliations:** 1 Medicine, Madras Medical College, Chennai, IND; 2 Internal Medicine, Madras Medical College and Rajiv Gandhi Government General Hospital, Chennai, IND

**Keywords:** coma, diabetic ketoacidosis (dka), dka, four score, gcs

## Abstract

Introduction

Altered mental status (AMS) is a critical presentation in diabetic ketoacidosis (DKA). Although the Glasgow Coma Scale (GCS) is the standard tool for evaluating consciousness, it has limitations when used in critically ill patients. The Full Outline of UnResponsiveness (FOUR) score, designed to address these, has been studied in neurocritical settings but remains underexplored in metabolic encephalopathies. This study aimed to compare the prognostic utility of GCS and FOUR scores in hyperglycemia-induced AMS.

Materials and methods

A cross-sectional pilot study was carried out at Madras Medical College involving 91 DKA patients with AMS. Baseline demographics, metabolic parameters, GCS, and FOUR scores were documented. Statistical analyses included Spearman’s correlation, regression models, and ordinal regression to identify associations between neuro-metabolic parameters and neurological outcomes. Ethical approval was obtained, and informed consent was secured from legally authorized representatives.

Results

Patients demonstrated severe metabolic derangements (mean glucose: 566 mg/dL, pH: 7.05, HCO₃-^:^ 10.9 mmol/L). GCS values were inversely proportional to anion gap (r=-0.211, p=0.044), while FOUR’s brainstem reflex component correlated positively with bicarbonate (r=0.30, p=0.004). Creatinine showed a protective association with GCS outcomes (odds ratio (OR)=0.55, p=0.040) and was positively correlated with the Brainstem reflex component of the FOUR score (rs=0.24, p=0.025). Overall, GCS reflected metabolic severity more strongly than FOUR, which showed limited associations.

Conclusions

GCS demonstrated stronger correlations with metabolic severity in DKA compared to FOUR, which showed only selective associations. Despite theoretical advantages, FOUR underperformed against GCS in this context. GCS should remain the primary tool for bedside neurological assessment in DKA, with FOUR serving as an adjunct in select cases. Larger multicenter studies are warranted to confirm these findings and evaluate integrated scoring approaches.

## Introduction

Altered mental status (AMS) is a broad clinical presentation encompassing disturbances in consciousness and cognition, often signalling underlying systemic or neurological pathology [1]. The Glasgow Coma Scale (GCS) is a tool used for the quantitative measurement of AMS in unconscious patients. GCS groups AMS into mild (GCS 13-14), moderate (GCS 9-12), and severely altered (GCS less than 8) [2]. Hyperglycemia is defined as a random capillary blood glucose greater than 200 mg/dL or 11.1 mmol/L [3]. Hyperglycemia in the form of diabetic ketoacidosis (DKA) or hyperosmolar hyperglycemic state (HHS) is a major metabolic cause of AMS [4]. Hence, accurate and timely assessment of consciousness in hyperglycemia-induced AMS is vital for prognostication and guiding therapeutic interventions.

The Full Outline of Unresponsiveness (FOUR) score is a validated, highly reproducible tool used to assess the level of consciousness across all populations, designed to address the limitations of the GCS [5]. Unlike the GCS, the FOUR score evaluates eye response, motor response, brainstem reflexes, and respiratory patterns, providing a more comprehensive neurological assessment, especially in intubated patients or those with severe metabolic derangements [6].

Although the FOUR score has been studied extensively in neurocritical care settings such as traumatic brain injury, stroke, and post-cardiac arrest coma, its application in metabolic causes of AMS, particularly hyperglycemia-induced states, remains underexplored. Existing literature predominantly relies on the GCS for assessing AMS, despite its known limitations in capturing subtle neurological and brainstem abnormalities, features captured by the FOUR score but not by the GCS. The limited research on the FOUR score highlights a critical gap in the literature: the lack of evidence on whether it offers superior prognostic accuracy in patients with metabolic encephalopathy.

This study aims to compare the associations of GCS and FOUR scores with the metabolic severity parameters. Moreover, establishing evidence in this area may encourage broader adoption of the FOUR score in metabolic encephalopathies, bridging a gap between neurocritical care and internal medicine practice. Ultimately, such an approach could standardize neurological assessment across diverse etiologies of AMS and enhance patient outcomes.

## Materials and methods

Ethical approval

The proposal for the present study was submitted to the Institutional Ethics Committee at Madras Medical College, Chennai, India, and received approval before the commencement of the research (approval number: 61042025, dated April 4, 2025). Ethical compliance was ensured throughout the study, with written informed consent obtained from the legally appointed representative (LAR) of all participating patients (usually a relative) in their native language.

Study design and setting

This was a cross-sectional study conducted from April to July 2025, among patients with hyperglycemia-induced altered consciousness due to diabetic ketoacidosis, admitted to the Institute of Internal Medicine, RGGGH, affiliated with Madras Medical College (MMC), Chennai, India. Other confounding conditions were ruled out by conducting investigations. 

Participant criteria

Participants included patients over 18 years of age presenting with hyperglycemia-induced altered mental state due to diabetic ketoacidosis. Altered consciousness is classified according to GCS scores: mild (GCS 13-14), moderate (GCS 9-12), and severe (GCS less than 8) [2]. Consent was obtained from the LAR or next of kin after explaining the study in the local language and emphasizing their right to decline participation. Individuals who were not willing to provide consent for their relatives were excluded from the study.

Sample size calculation

Since the present work was conducted as a pilot study, the sampling strategy aimed to include all eligible cases available during the study period. This comprehensive approach ensured that the study captured the maximum number of cases accessible at the time, resulting in a final sample size of 91.

Data collection

For all participants, comprehensive baseline demographic and clinical data, like age, sex, and capillary blood glucose level, were collected. Following consent, for each patient, the GCS score was calculated by a trained junior resident based on eye, verbal, and motor responses. The FOUR score was calculated separately using assessments of eye response, motor response, brainstem reflexes, and respiration, which were documented. Ionic parameters and anionic gap metrics were also noted from the patient charts for comparative analysis. The data were recorded on Google Sheets to undergo further analysis.

Data analysis

Data were analyzed using SPSS Statistics software version 24 (IBM Corp., Armonk, NY) and Microsoft Excel. Continuous variables are presented as means ± standard deviation (SD) with 95% confidence intervals (CIs), and categorical variables as counts and percentages. DKA severity was compared via the Kruskal-Wallis test. Associations among neuro‑metabolic parameters were examined using Spearman’s correlation. Ordinal regression models evaluated relationships between neuro‑metabolic severity and GCS and FOUR scores (and their components). Assumptions for all models were checked. Significance was set at p<0.05; effect sizes are reported as correlation coefficients, regression β’s, and odds ratios (ORs).

## Results

Study population and baseline characteristics

A total of 91 patients with DKA were included in the analysis, as no patients with HHS were admitted to the hospital during this period. The study population comprised 42 males (46.2%) and 49 females (53.8%), with a mean age of 65.7 ± 15.0 years (95% CI: 62.6 - 68.9). The majority of patients were older adults, with 48 patients (52.7%) aged ≥65 years and 43 patients (47.3%) aged <65 years (Table 1).

**Table 1:** Demographic characteristics of the study population SD: standard deviation; FOUR: Full Outline of Unresponsiveness; GCS: Glasgow Coma Scale

Characteristic	Value	Range
Age, years, mean ± SD	65.7 ± 15.0	62.6 – 68.9
Male gender, n (%)	42 (46.2%)	
<65 years, n (%)	43 (47.3%)	
Capillary blood glucose, mg/dL, mean ± SD	566.1 ± 192.5	526.0 – 606.2
pH, mean ± SD	7.05 ± 0.11	7.03 – 7.08
Bicarbonate, mmol/L, mean ± SD	10.9 ± 4.6	9.9 – 11.8
Chloride, in mmol/L, mean ± SD	101.6 ± 5.4	100.4 – 102.8
Sodium, in mmol/L, mean ± SD	138.9 ± 7.4	137.4 – 140.4
Potassium, in mmol/L, mean ± SD	4.5 ± 0.8	4.4 – 4.7
Anion gap, mmol/L, mean ± SD	27.3 ± 7.1	25.9 – 28.8
Creatinine, mg/dL, mean ± SD	2.04 ± 0.84	1.86 – 2.22
FOUR score, mean ± SD	7.9 ± 2.6	7.3 – 8.5
GCS score, mean ± SD	9.6 ± 2.4	9.1 – 10.1

Metabolic Parameters

The cohort demonstrated severe metabolic derangements. The mean capillary blood glucose was markedly elevated at 566.1 ± 192.5 mg/dL (95% CI: 526.0 - 606.2). Metabolic acidosis was confirmed by a mean pH of 7.05 ± 0.11 (95% CI: 7.03 - 7.08) and bicarbonate level of 10.9 ± 4.6 mmol/L (95% CI: 9.9 - 11.8). The mean anion gap was elevated at 27.3 ± 7.1 mmol/L (95% CI: 25.9 - 28.8), consistent with high anion gap metabolic acidosis. Females demonstrated significantly higher anion gap values compared to males (median 30.9 vs 26.8 mmol/L, U=713.5, p=0.012, r=0.26). Renal function was impaired, with mean creatinine levels of 2.04 ± 0.84 mg/dL (95% CI: 1.86 - 2.22).

Neurological Assessment Scores

Neurological function was significantly impaired across both assessment scales. The FOUR score averaged 7.9 ± 2.6 (95% CI: 7.3 - 8.5), while the GCS score was 9.6 ± 2.4 (95% CI: 9.1 - 10.1), indicating moderate to severe neurological dysfunction in the study population.

Neurological-metabolic correlations

**Figure 1:**
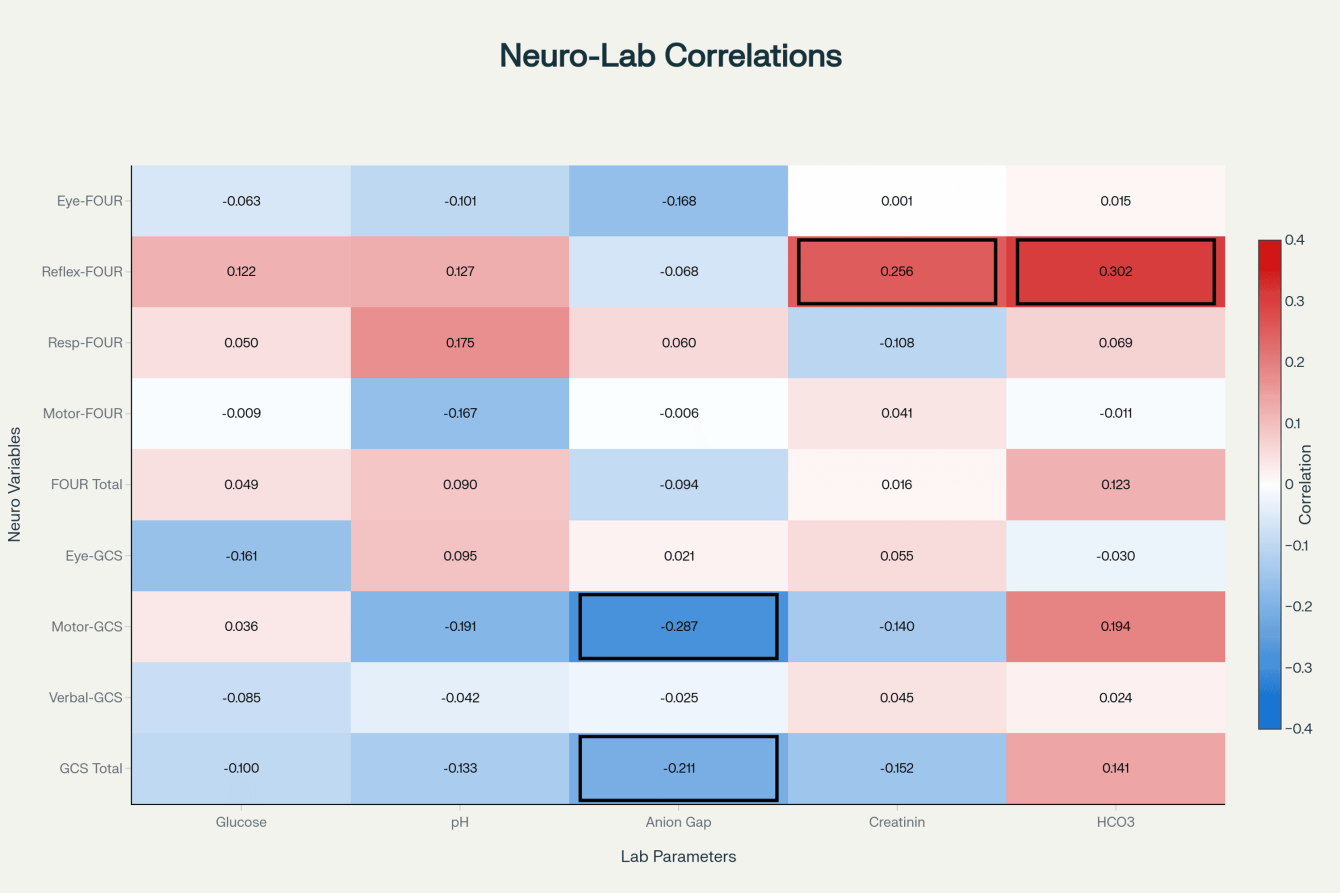
Neurological-metabolic correlations Figure showing the correlation between the neurological parameters (components of the scores) and the laboratory investigations FOUR: Full Outline of Unresponsiveness; GCS: Glasgow Coma Scale

Correlation analysis identified several significant associations between metabolic parameters and neurological outcomes. Bicarbonate (HCO_3-_) demonstrated the strongest positive correlation with the brainstem reflex component of the FOUR score (rs=0.30, p=0.004). Anion gap showed significant negative correlations with neurological function, specifically with the motor component of GCS score (rs=-0.29, p=0.006) and overall GCS scores (rs=-0.21, p=0.044). Creatinine levels were positively correlated with the brainstem reflex component of the FOUR score (rs=0.24, p=0.025). No significant correlations were observed between capillary blood glucose or pH and any neurological assessment parameters (Figure 1).

Severity classification analysis of DKA

Based on serum bicarbonate levels, serum pH and Anion gap patients were stratified into metabolic acidosis severity groups: severe acidosis (HCO_3-_: 3.1 - 9.3 mmol/L) in 43 patients (47.3%), moderate acidosis (HCO_3-_: 10.5 - 14.9 mmol/L) in 29 patients (31.9%), and mild acidosis (HCO_3-_: 15.1 - 18.0 mmol/L) in 19 patients (20.9%). To minimize atypical or mixed severity classification, serum bicarbonate levels are taken as a key parameter for metabolic grading of acidosis.

Severity analysis across mild, moderate, and severe graded groups revealed significant differences in the motor component of the FOUR scores (H=7.69, p=0.021, η²=0.065). Post-hoc analysis revealed an unexpected U-shaped pattern in the motor component of FOUR scores across severity groups: severe group (1.95 ± 1.34, median 2.0), moderate group (1.28 ± 1.39, median 1.0), and mild group (2.32 ± 1.34, median 3.0). No significant differences were observed in other neurological parameters across metabolic severity groups.

**Figure 2:**
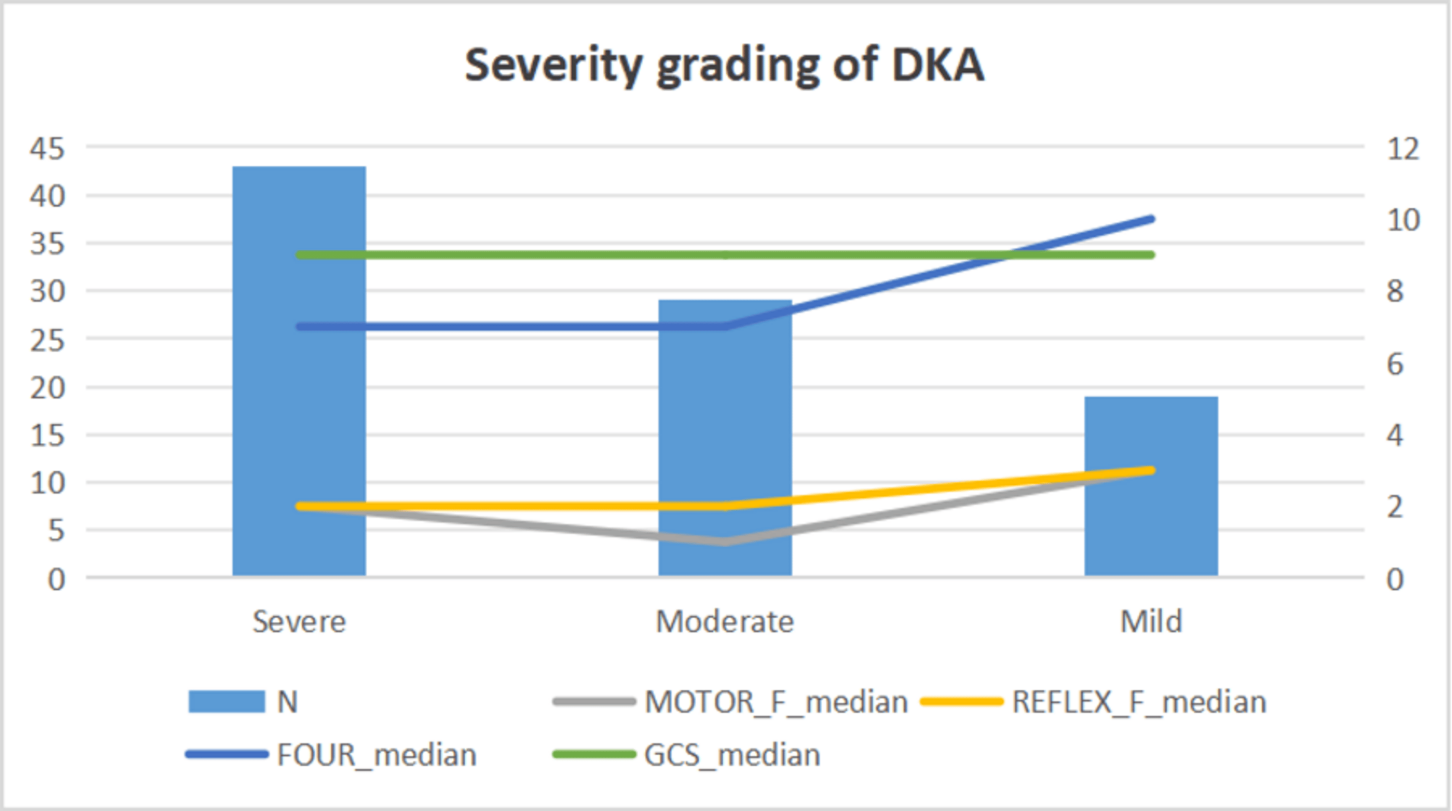
Severity classification of DKA Figure depicting the severity of DKA and its correlation with the components of FOUR and GCS DKA: diabetic ketoacidosis; FOUR: Full Outline of Unresponsiveness; GCS: Glasgow Coma Scale

A severity grading analysis was performed based on blood glucose level: mild hyperglycemia (250 - 400 mg/dL) in 22 patients (24.2%), moderate hyperglycemia (400 - 600 mg/dL) in 26 patients (28.6%), and severe hyperglycemia (>600 mg/dL) in 43 patients (47.2%). Significant difference was observed in serum creatinine level (H=6.08, p=0.048, η²=0.045). The pairwise comparisons revealed a trend toward higher creatinine levels in both moderate and severe groups compared to the mild group, though these differences were not significant after Bonferroni correction for multiple testing (p>0.05). However, no other significant differences were observed in the neurological scores (Figure 2).

Predictive modeling results

Several significant predictors were identified for different outcomes. For poor GCS outcomes, creatinine (β=0.085, p=0.030) was a significant predictor. Acidosis was significantly predicted by GCS score (β=0.022, p=0.045). No significant outcome for a poor FOUR score was observed.

**Figure 3:**
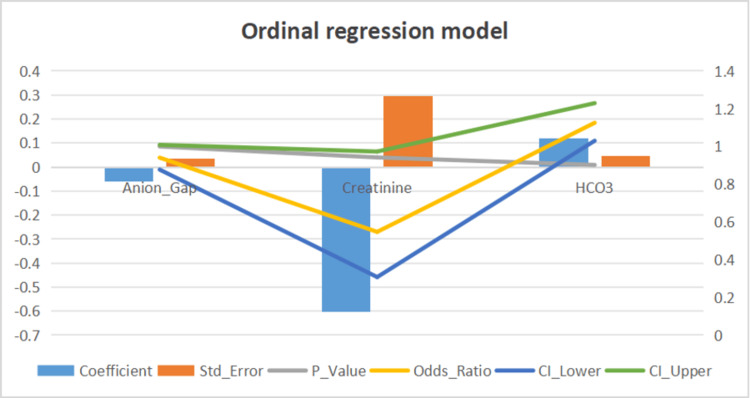
Ordinal regression model Regression model depicting the anion gap, creatinine, and HCO_3_

Ordinal regression models for neurological scoring outcomes identified several significant predictors. For GCS grades, serum creatinine showed a protective effect (OR=0.55, 95% CI: 0.31-0.97, p=0.040). while non-significant trends showed that lower pH trended towards lower GCS scores (p=0.093) and larger anion gap tended to lower GCS (p=0.086). The FOUR grade analysis revealed that bicarbonate levels had a positive association with better outcomes (β=0.075, p=0.137). Brainstem reflex component of the FOUR score was significantly associated with serum bicarbonate levels (β=0.118, p=0.009), consistent with the correlation analyses (Figure 3).

## Discussion

Diabetes mellitus is a major global health problem, and DKA is one of its most severe acute complications [7]. DKA is characterized by uncontrolled hyperglycemia, metabolic acidosis, and electrolyte imbalance, and frequently presents with AMS, ranging from mild confusion to deep coma. AMS in the setting of DKA carries significant prognostic implications and requires prompt and accurate assessment for appropriate management [8]. The GCS has long been the most widely used clinical tool for evaluating consciousness. However, it has notable limitations, particularly in patients who are intubated or unable to provide a verbal response [9]. To address these shortcomings, the FOUR score was developed, incorporating domains such as brainstem reflexes and respiratory patterns [10]. Theoretically, the FOUR score provides a more comprehensive neurological assessment, especially in critically ill patients, and is considered slightly superior to GCS in settings where verbal assessment is not feasible [11].

In the present study, the correlation between the FOUR score components and the collected values yielded no significant findings, except for the brainstem reflex component and HCO_3-_ values, which showed an r value of +0.302. This indicates that worsening acidosis can suppress brainstem reflexes. The brainstem reflex component and pH had a mild positive correlation of +0.127, which brings us to the same conclusion. The GCS scores had a high negative correlation with the anion gap, having an r value of -0.211. These stronger negative correlations in GCS as compared to the FOUR score indicate that the GCS outperforms the FOUR score in practice, despite FOUR being theoretically better.

The GCS is frequently cited in the literature and is widely regarded as the gold-standard tool for assessing AMS [12]. The primary studies that compared FOUR and GCS were in the context of traumatic brain injury, where the FOUR score proved to be better [13]. Perhaps the best indication for the usage of the FOUR score seems to be hepatic encephalopathy [14]. Although studies have used the GCS as the gold standard in patients with DKA, there appears to be no existing literature directly comparing these scoring systems in the context of DKA [15]. As a pilot study, there is also limited literature exploring the use of the FOUR score in patients with DKA. Additionally, the FOUR score has been utilized to assess outcomes in patients following cardiac arrest [16].

The clinical implication of these findings is that the GCS should remain the primary bedside tool for neurological assessment in DKA, while the FOUR score may serve as a useful complementary measure, particularly in specific clinical situations. Nevertheless, the present study underscores the importance of evaluating newer scoring systems against established standards, and further research with larger patient populations and prospective designs would be valuable to confirm these findings and refine the role of the FOUR score in hyperglycemia-related emergencies.

The present study has certain limitations. The sample size was relatively small and derived from a single center, which may limit the generalizability of the findings. Furthermore, the data were limited to patients with DKA only; therefore, the findings may not apply to other causes of hyperglycemia-associated altered consciousness, such as hyperosmolar hyperglycemic state. The negative correlation observed between GCS and pH (r = -0.133) may reflect variability or noise in the sample data. Additionally, certain clinical variables that may influence outcomes - such as duration of diabetes, presence of comorbidities, treatment variations, and other confounding factors like infections - were not incorporated into the analysis. Finally, as with any single-center study, external validation across diverse clinical settings is necessary before definitive conclusions can be drawn regarding the comparative predictive accuracy of the GCS and FOUR scores.

## Conclusions

This study demonstrates that the FOUR score showed only a mild positive correlation between brainstem reflexes and pH/HCO₃⁻ values, whereas the GCS demonstrated a stronger negative correlation with the anion gap, suggesting that GCS better reflected metabolic severity in DKA. Creatinine levels were associated with better GCS outcomes, indicating a protective effect. They also showed a positive correlation with the brainstem reflex component of the FOUR score. The literature predominantly supports the use of GCS for assessing AMS, while the advantages of the FOUR score have been mainly observed in traumatic brain injury and hepatic encephalopathy, with limited research exploring its role in DKA. This study highlights that, despite the theoretical advantages of the FOUR score, GCS seemed to be better than the FOUR score in this setting. These findings suggest that GCS should remain the primary tool for bedside neurological assessment in DKA, while the FOUR score may serve as an adjunct in specific clinical situations. Future research involving larger, multi-center studies is needed to validate these observations and explore whether integrating both scoring systems could enhance accuracy in evaluating hyperglycemia-related neurological dysfunction.
